# Arrow Wounds*Extract from an address read before the Buffalo Medical Club at its annual meeting, May 11, 1881.

**Published:** 1881-07

**Authors:** H.S. Kilbourne

**Affiliations:** Captain and Asst. Surgeon, U. S. A.


### ARROW WOUNDS.*

BY H. S. KILBOURNE, M. D., CAPTAIN AND ASST. SURGEON, U. S. A.

THERE are, gentlemen, some peculiar features of military surgery, one of which I will take up briefly for your entertainment this evening. I say for your entertainment, because the special feature of which I now speak will be, to you, more curious than practical. It is characteristic of a people and a period now changing.

It belongs, almost exclusively, to military surgery and is, even now, a subject with which many military surgeons are unfamiliar.

It is one not treated of in systematic works on surgery. In many of these it is not mentioned. Its literature belongs mostly to the historical period, before the days of gunpowder. I allude to arrow wounds.

You, whose hearts have been touched by Greek fires, may remember that of all the heroes at the siege of Troy, whose wounds and deaths in battle are chronicled by the past, many are killed by arrows; and of these, scarcely two are struck in the same place, or die in the same way.

The godlike Achilles, in order to become invulnerable, was plunged by his goddess mother into the river Styx. But as she held him by the heel, that part only was not bathed by the Stygian flood. There, finally, the arrow shot by Paris found entrance. Hippocrates, in his numerous campaigns, invented or used an instrument for the extraction of arrows and darts, called *belulcrum*—a rude forcep for seizing and removing rude missiles from wounds.**

As arrows were among the first offensive weapons used by man in ancient and barbaric times, so they are still the favorite weapon of many savage tribes. This antique weapon is, however, being rapidly displaced by rifles among those tribes whose

* Extract from an address read before the Buffalo Medical Club at its annual meeting, May 11, 1881.

** Dr. Otis, U. S. A., in Circular No. 3, S. G. O.

enmities with the whites have taught them its superiority. An American Indian of the plains, who has seen a breech loader and does not possess one, will part with all he has in the world to procure it. The bow and arrow is therefore disappearing, and with them the surgery of arrow wounds will become only a curious history.

The service bow of the Indian is quite a different thing from the dainty affair of the Archery clubs. It is short, thick and strong. It is made among the southwestern tribes of the bois d'arc or Osage range. The string is of raw hide, or buffalo sinew. The arrow is made of pecan or any suitable wood, not too heavy and having a straight, tough fibre. The arrow head, formerly of flint, is now usually made of soft iron, cut by a file into triangular shape. It is let into the split end of the shaft by a short shank and fastened by wrapping with strips of sinew, put on before it is dry.

In hunting and fighting, the warrior is mounted on a swift and active pony, and in using the bow he comes to close quarters. In action he rides at full speed, slipping over to the side of the horse and discharging the arrows from under the neck of the animal. The horse thus acts as a living shield for his body.

The rapidity, force and precision of the shots is, in some instances, marvelous. As a rule, however, the accuracy is about that of a fair marksman with the pistol, as at a running mark.

There is in the Army Medical Museum, at Washington, a preparation of a portion of the left scapula of a buffalo, with an arrow head impacted in it. The barbed iron head of the arrow has entered the venter and the point protrudes from the dorseum, so that the missile must have passed through the thorax.

The specimen is from a buffalo killed at Fort Sedgwick, in 1860, by a Cheyenne Indian. There are also other specimens of bones, penetrated, perforated and fissured in various ways, some of them resembling the appearance of glass, through which a pistol ball has been found a few yards off*. The rapidity of

*Dr. Otis, U. S. A., in Report in Surg. Cases in the Army.

the discharge of arrows rival the fire of the first breech-loader I know of. The precision is sufficient to make it exceedingly uncomfortable for any one in the vicinity.

As the arrow head is secured to the shaft by strips of dried sinew, the wounds inflicted by it have this peculiarity: that in penetrating wounds, the fastening is softened by the fluids of the body, and when not at once extracted, the head becomes loosened and is detached when extraction is attempted.

In such cases it remains as a foreign body in the wound. If bone has been penetrated, the arrow head is impacted in it and held fast by the compact tissue, when extraction may become a difficult and hazardous operation.

The late Gen. Kearing suffered from a penetrating arrow wound of the superior maxillary bone, received in an affray with hostile Indians, about 1855 or 1856, until his death. The arrow head was so pinched and twisted by the hard bone, and attempts at removal were attended by such copious hæmorrhage, that the iron was allowed to remain until a secondary operation was practicable. The missile was finally removed in St. Louis, after a severe operation, but the patient never entirely recovered from its effects. At short range, the arrow will perforate a flat bone, as a rib, making a clean cut, or a slight cracking or splitting. There is seldom any comminution or extensive fissuring like that produced by the impact of a conoidal rifle ball with bone.

In all the specimens showing penetration of the cranium, there is, according to Dr. Otis, little or no fissuring internally or externally. The inner and outer tables are both cleanly divided. This feature distinguishes arrow wounds of the calvaria from all others caused by weapons used in civilized warfare. Indeed, among all weapons I have seen, I know of none capable of making wounds so peculiar.

There are, in the army museum, specimens illustrating these features and also the impaction of arrow heads.

For the relief of these injuries, it is most natural to seize the arrow and draw it out of the wound. But that will never do. The head will part with the shaft and remain in the bottom of the wound.

If not loosened by softening of the sinew, the head, if barbed, will catch in the soft parts and may, in certain regions, work irreparable injury.

Dr. Bill, of the army, has employed a device for these operations which is often of great service. He passes the loop of a wire snare down along the shaft and over the arrow head, and when in place, secures it by traction on the outer end of the loop. The missile may then be carefully withdrawn entire. When the iron is impacted in the bone, he employs a loop of annealed wire, passed down along the shaft, which serves as a guide. The arrow head is snared by the loop, the ends of the wire are then passed through a long suture twister, or double canula, which is passed down to the loop; the ends of the wire are then twisted or secured to the handle, and arrow and instrument are withdrawn together.

In a case where a Navajo arrow had penetrated the lung to a depth of five inches, Dr. Bill removed it by means of the simple snare first described.

A prominent army officer, now retired from active service, when serving in Texas as a subaltern, was, while in an encounter with hostile Comanches, transfixed by an arrow.

The weapon pierced the upper part of the right chest and passed nearly horizontally through the lung, the point protruding at the back, between the scapula and spine, on the same side. He informed me that at his own request a silk handerkerchief was fastened to the shaft, which was then pushed on through the body, dragging the silk after it through the whole extent of this formidable wound.

He recovered from the effects of the wound and from this novel surgical procedure, and served actively for many years after.

In the report of surgical cases in the army since the war, I find the following somewhat similar case, reported by Dr. Goddard, U. S. A. His employee at Fort Rice, D. T., was wounded in February, 1868, by an arrow, which entered his back three inches to the right of the fifth lumbar vertebra and emerged at a point two inches to the right of the ensiform cartilage. During the following evening the patient lost, externally, about eight ounces of blood and a small (estimated) quantity, internally. He was confined to his bed some two weeks, suffering from irritative fever and circumscribed peritonitis. In four weeks he was walking about and on July 1st was actively employed. There are no further particulars or comments on this case.

The route of the missile is a mystery. It would seem impossible that it could have been direct between the two wounds, as the intestines, stomach and liver lie in the way. Both the great cavaties would also have been opened. Penetrating arrow wounds of the abdomen are usually mortal.

Of 9 cases reported in detail, 7 were fatal; and of the 2 which recovered, it is doubtful whether the peritoneal sac was penetrated. One of the fatal cases was mine, and as it may be taken as a type of its class, I will give it here: A cavalry soldier was wounded while approaching an Indian camp at night, near the Canadian river, Texas, by an arrow which entered the abdomen in the left hypochondriac region, making a wound about three-fourths of an inch in length, throngh which about eighteen inches of the small intestine protruded. The gut was cut in four places. The wounds in the intestines were closed by sutures and the protruding portion returned through the wound, which was enlarged for the purpose. When found, the man had lain out all night, and was in a state of collapse. He was carried along with the column, in an ambulance, but died on the second day, never having rallied from the shock of the injury.

The great fatality of arrow wounds of the abdomen is so well known to the Indians that in action they usually aim at the umbilicus.*

*Dr. Bill, U. S. A.

Another case, which I personally observed, is so unique that I will briefly give its outlines. It is an example of a penetrating arrow wound of the lower part of the trunk. The operation for its relief was done at Fort Sill, I. T., by Dr. Forwood, of the army, who reports the case. I assisted him and saw the patient up to the date of his removal.

"Latimore, a chief of the Kiowas, aged 42 years, applied at Fort Sill for treatment, with symptoms of stone in the bladder. In 1862 he had led a band of his tribe against the Pawnees, and was wounded in a fight. Being mounted and leaning over his horse, a Pawnee, on foot and within a few paces, drove an arrow deep inte his right buttock. The shaft was withdrawn by his companions, but the point remained in his body. He passed bloody urine soon after the injury, but the wound soon healed, and in a few weeks he was able to ride without inconvenience. For more than six years he continued at the head of his band and travelled on horseback many hundreds of miles every summer. A long time after the injury he began to feel pain in urination, which increased until he was forced to reveal the sacred secret, as it is regarded by the Indians, and to seek medical aid. The urine was loaded with blood, mucus and pus; the introduction of a sound indicated a large, hard calculus in the bladder. Judging from the cicatrix and all the circumstances, it was apparent that the arrow had passed through the glutic muscles and the obturative (sciatic?) foramen into the bladder. On Aug. 23d I removed the stone by the lateral operation. The calculus was phosphatic, and weighed eight hundred and fifteen grains. On section of the stone, the arrow point was found imbedded near its centre. It was of iron and had originally been about two and one-half inches long by seven-eights of an inch broad, in its widest part. The urine began to pass by the natural channel on the third day, and on the seventh day it had nearly ceased to flow from the wound. But the restless spirit of the patient's band could no longer be restrained; open hostilities with the whites were expected to begin every moment, and they insisted on his removal. He needed purgatives on the eighth day, which they refused to allow him to take; on the following day they started with him to their camp, 60 miles away. Fourteen days after he is reported to have died. But his relatives have since assured me that the wound had healed, and that no trouble arose from it."

Notwithstanding a narrow pelvic outlet and the large size of the stone, there was every reason to expect a favorable result in this interesting case. These Indians possess remarkable powers of endurance and recuperation from wounds, due, doubtless, to their active life in the open air of the Western plains. But it is possible that the case may have been complicated by an induced disease of the kidneys.

They have in New Mexico a diet of Spanish origin, composed of meats of different kinds and vegetables of various sorts, and spices—mainly pepper; and this is served for a dinner, breakfast, or supper, as the case may be—and that is all there is. It is a good dish for a hungry man, but would not delight an epicure. It is called *olla podrida*, or, more simply, *olla*. One who does not like meat, would eat it for the sake of the vegetables and the flavor they have imparted; and one whose fancy turned away from the vegetables, might take them on account of the meat in the essence of which they had been steeped; while he who would take neither flesh nor grass, might help himself to pepper.

Mr. President and gentlemen, it is an *olla* that I have set before you to-night.

